# Griffiths phases in infinite-dimensional, non-hierarchical modular networks

**DOI:** 10.1038/s41598-018-27506-x

**Published:** 2018-06-14

**Authors:** Wesley Cota, Géza Ódor, Silvio C. Ferreira

**Affiliations:** 1 Departamento de Física, Universidade Federal de Viçosa, 36570-000 Viçosa, Minas Gerais Brazil; 2 MTA-EK-MFA, Centre for Energy Research of the Hungarian Academy of Sciences, H-1121 P.O. Box 49, Budapest, Hungary; 3 National Institute of Science and Technology for Complex Systems, Rio de Janeiro, Brazil

## Abstract

Griffiths phases (GPs), generated by the heterogeneities on modular networks, have recently been suggested to provide a mechanism, rid of fine parameter tuning, to explain the critical behavior of complex systems. One conjectured requirement for systems with modular structures was that the network of modules must be hierarchically organized and possess finite dimension. We investigate the dynamical behavior of an activity spreading model, evolving on heterogeneous random networks with highly modular structure and organized non-hierarchically. We observe that loosely coupled modules act as effective rare-regions, slowing down the extinction of activation. As a consequence, we find extended control parameter regions with continuously changing dynamical exponents for single network realizations, preserved after finite size analyses, as in a real GP. The avalanche size distributions of spreading events exhibit robust power-law tails. Our findings relax the requirement of hierarchical organization of the modular structure, which can help to rationalize the criticality of modular systems in the framework of GPs.

## Introduction

A recurrent feature of complex systems is the presence of critical states, in which spatial and temporal correlations diverge^1,2^. A fundamental question is why and how a complex system would be tuned to criticality^3–5^. As a very important example, recent experimental evidences suggest that the brain operates near criticality^5–8^. Information processing capabilities, sensitivity and the dynamic range of stimuli, where the collective response varies significantly, are optimal in this region^9–12^. Simple models on homogeneous substrates^5,13^ have frequently been used to answer this question and criticality is often associated with some self-organization^3^ or evolutionary selection mechanism^14^. However, heterogeneity of the networks mediating the interactions among the agents of a dynamical process can be relevant for the outcomes of models investigated on them^15–17^, in particular, the quasi-static (quenched) disorder, with timescales much longer than those of the dynamics. Thus, it is a challenge to understand how quenched disorder originated from the heterogeneous network topology affects the observed critical state.

**Figure 1:**
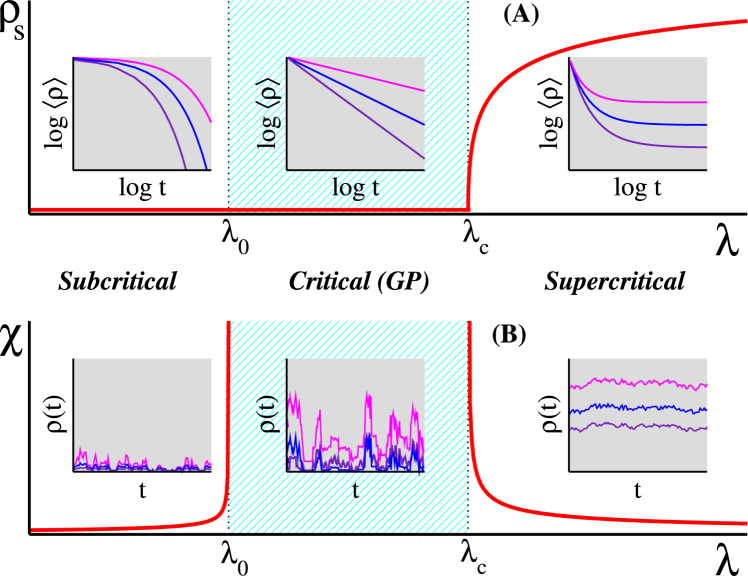
Griffiths phases and dynamical criticality in spreading processes. The top panel shows the stationary order parameter *ρ*_s_, which is the average density of active vertices, against the control parameter *λ*. The order parameter has a finite value above the critical point *λ*_c_ and vanishes as *λ* → *λ*_c_. The stationary density is zero for *λ* < *λ*_0_. In both regimes, the characteristic times to reach the stationary state are finite, typically given by exponential decays in the curves of average density against time. The asymptotic density is still null in the critical region, but the approaching to the asymptotic value is slow, typically a power-law time decay, manifested by straight lines on log-log plots. The bottom panel shows the stationary dynamical susceptibility^29,30^, defined as the relative variance of the order parameter, given by *χ* = *N*[〈*ρ*^2^〉 − 〈*ρ*〉^2^]/〈*ρ*〉, against the infection rate under the presence of a small external stimulus, which can be a spontaneous self-activation^65^. Sub and supercritical phases present finite susceptibility diverging as we approach the critical region, in which it remains infinite. The fluctuations are finite in the off-critical interval and huge within the whole critical region, representing high sensitivity to external stimuli. The subcritical, critical and supercritical regimes are schematically represented in the left, center, and right insets, respectively.

In condensed matter physics, quenched disorder can lead to the so-called Griffiths phases (GPs)^18^ with dynamical criticality and high sensitivity to external stimuli in an extended parameter space^19^. Dynamical criticality means long-term temporal correlations that imply slow relaxation and broad distributions of interevent times manifested usually as power laws (PLs)^2,20^. GP is the consequence of rare regions (RRs), consisting of local (sub-extensive) supercritical (active) domains, which occur with small probability but sustaining activity for long times (exponential in domain size). To understand GPs in critical dynamics, consider a dynamical spreading process with active and inactive states, a control parameter *λ* and an order parameter *ρ* (density of active individuals) determining the system phase. Inactive states are also called “absorbing” because, once visited, no other state can be reached from it without an external source^20^. The system is in a globally active phase for *λ* > *λ*_c_, with a non-zero order parameter as *t* → ∞, or in an inactive one, for *λ* < *λ*_0_ with *ρ* = 0, for *t* → ∞. Long lived RRs are absent in the latter case^19^. Response functions, which quantify the sensitivity to external stimuli, are finite in both ranges; see Fig. 1. In the interval *λ*_0_ < *λ* < *λ*_c_, the activity in RRs is long lived, but ends up due to the fluctuations in the finite sized local patches. Convolution of low-probability RRs and exponentially long lifetimes results in a slow relaxation and highly fluctuating dynamics characterized by nonuniversal exponents in this interval, constituting a GP^19^. Response functions become very large (formally infinite in the thermodynamic limit) in this range and the system exhibits hypersensitivity to external stimuli. Figure 1 shows a scheme for GPs and dynamical criticality. A central question is if the RR effects are strong enough to alter the phase transition^21^.

Critical systems can be sensitive to quenched disorder when their dimension is sufficiently low^19^. A hypothesis based on activity spreading models claims that the heterogeneity effects become irrelevant in the thermodynamic limit in case of infinite-dimensional random graphs^22^. The dimension *d*_*g*_ of a graph is given by the relation between the average number of nodes ${\mathscr{N}}$ enclosed in a distance *l*, such that ${\mathscr{N}}\sim {l}^{{d}_{g}}$. For small-word networks, for which distances increase logarithmically with the graph size^23^, we formally have *d*_*g*_ = ∞. Some evidences suggest that the brain network is heterogeneous and present hierarchical modular organization, in which modules are themselves composed by modular substructures at distinct levels^10,24,25^. These inspired Moretti and Muñoz^26^ to investigate activity spreading models on hierarchical modular networks of finite dimension, for which GPs and extended critical regions were observed (see also^27,28^) and to conjecture that the brain criticality could be effected by quenched disorder without fine parameter tuning. It does not mean that one cannot find relevant effects in finite non-modular systems^29–31^. Moreover, long-range connections can drastically increase the network dimension, even if they constitute just a small portion of the graph^32,33^. The empirical organization of biological networks is highly complex and subjective^10^ and, therefore, it is not completely clear whether real brain networks in a cellular level are actually hierarchical^34^. Furthermore, modular graphs without hierarchical structure are observed in diverse important systems such as socio-technological^35,36^ or protein interaction networks^37^, but the existence of extended critical regions due to the quenched disorder on such systems has not been considered extensively.

To our knowledge, no investigation has been done to scrutinize whether hierarchy is really a necessary condition for the emergence of GPs. The present work aims at to fill this gap, using simulations of activity spreading models on non-hierarchical modular structures. Recently, optimal fluctuation theory^31^ and simulations provided extended critical regions on heterogeneous networks of finite size constrained to averages over independent network samples^30^. This inspired us to investigate the dynamical behavior of the continuous time Markovian susceptible-infected-susceptible (SIS) model, which has been used to describe activity or information spreading in socio-technological and biological systems^26,38–40^, on loosely coupled network of modules. We found extended control parameter regions with non-universal PL decays of activity in time, which are size-independent, calling for the existence of real GPs in infinite dimensional, but loosely connected modular structures. Thus, our results point out that we can relax the requirement of hierarchical organization and large-world^22,26^ for the existence of GPs on modular networks, although these factors certainly enhance RR effects.

## Results

### Synthetic modular networks

**Figure 2:**
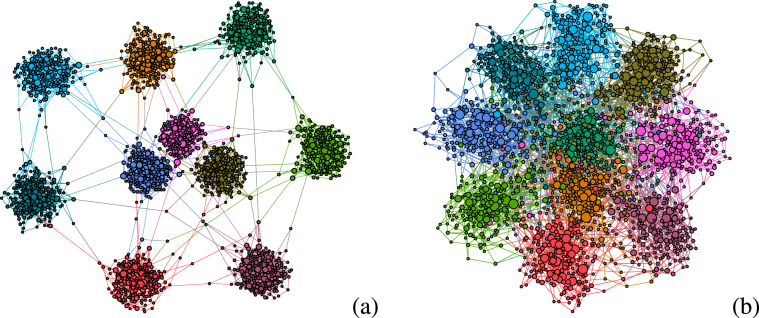
Examples of modular structures. Networks with *M* = 10 modules of same size *S* = 200 and number of intermodular connections (**a**) ${k}^{[{\rm{out}}]}=10$ and (**b**) 100 representing loosely and densely connected modular graphs, respectively. The network degree distribution is given by $P(k)\sim {k}^{-2.7}$ with ${k}^{[{\rm{low}}]}=3$ and ${k}^{[{\rm{upp}}]}=14$ for the lower and upper bounds cutoffs, respectively. Connected modular structures can clearly be observed. Nodes in a same community are plotted with the same color and their sizes are proportional to the vertex degree. The graph was generated using Gephi visualization tool (https://gephi.org).

We generated modular networks based on the benchmark model of Lancichinetti, Fortunato, and Radicchi^41^. Consider *g* = 1, …, *M* modules where the size *S*_*g*_ of each group is drawn according to a distribution *Q*(*S*_*g*_). At a vertex level, the degrees are drawn from a distribution *P*(*k*) with $k={k}^{[{\rm{low}}]},\ldots ,{k}^{[{\rm{upp}}]}$ where ${k}^{[{\rm{low}}]}$ and ${k}^{[{\rm{upp}}]}$ are lower and upper cutoffs of the degree distribution, respectively. The maximal number of *intermodular* edges connecting vertices of different groups is predefined as ${k}_{g}^{[{\rm{out}}]}$ and, in general, can depend on the module. By construction, this model produces highly modular networks if the number of intermodular connections is much smaller than the *intramodular* one, which was confirmed by the calculation of the modularity coefficient^42^ and using the Louvain community detection algorithm^43^. See section Methods for network metrics and generation procedure. Figure 2 shows examples of modular networks with different levels of intermodular connectivity.

Depending on the module size distribution *Q*(*S*_*g*_), we divided the investigated networks into two classes. In the monodisperse modular networks (MMNs), all modules have exactly the same number of vertices and of intermodular connections, *i*.*e*. *S*_*g*_ = *S* and ${k}_{g}^{[{\rm{out}}]}={k}^{[{\rm{out}}]}$ for *g* = 1, …, *M*. However, real modular networks are not monodisperse in the aforementioned sense and, thus, we also considered a PL distribution of module size with $Q({S}_{g})\sim {S}_{g}^{-\varphi }$, which are consistent with observations in real systems^41^. The upper bound of the distribution is limited to the system size (*S*_*g*_ ≤ *N*) while the lower one is chosen such that the average module size 〈*S*_*g*_〉 has a predefined value. These networks are referred hereafter as polydisperse modular networks (PMNs). We also chose the number of intermodular connections proportional to the module size, ${k}_{g}^{[{\rm{out}}]}\propto {S}_{g}$, constraining ${k}_{g}^{[{\rm{out}}]}\ge 2$, to guarantee connectivity^23^. We used the values of 〈*S*_*g*_〉 = 10^3^ and $\langle {k}_{g}^{[{\rm{out}}]}\rangle =5$ in all presented results to perform comparison between monodisperse and polydisperse cases. Brain networks, which inspired our research, are not fully random like those presented here, but our aim was to isolate the role played by the network dimensionality.

**Figure 3:**
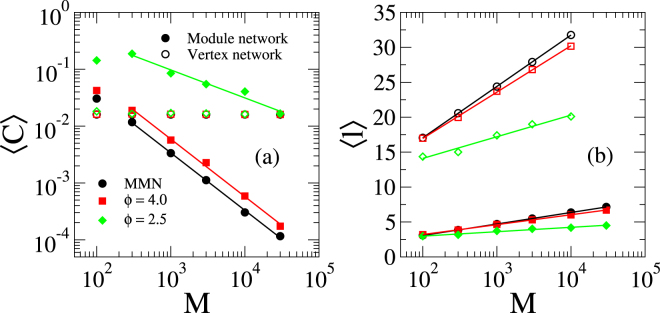
Structural properties of modular networks. (**a**) Clustering coefficient and (**b**) average shortest path as functions of the number of modules. Open symbols correspond to the vertex network, while the filled ones represent the network of modules, in which the modules are themselves treated as nodes connected by the intermodular edges. Lines denote (**a**) power-law or (**b**) logarithmic regressions. In monodisperse modular networks (MMN), all modules have the same number of vertices *S* = 〈*S*_*g*_〉. Networks with module size distribution $Q({S}_{g})\sim {S}_{g}^{-\varphi }$ are obtained by fixing the minimal size *S*_min_ such that the chosen average size is obtained. The parameters are $\langle {k}_{g}^{[\mathrm{out}]}\rangle =5$, 〈*S*_*g*_〉 = 10^3^, *γ* = 2.7, ${k}^{[\mathrm{low}]}=3$, and ${k}^{[\text{upp}]}=58$; see main text. The averages were performed over 25 independent networks.

We determined the average clustering coefficient and the average shortest mean distance^23^ for both vertex and module networks. The latter means that we treat modules as vertices, connected by intermodular edges forming a network. Structural properties of these modular networks are shown in Fig. 3. The clustering coefficient, averaged over the whole network, saturates at a small finite value as the network size increases (see Fig. 3(a)). This is a natural consequence of the modular organization of the network that forces vertices to be connected mostly within the modules which are of finite size and thus the probability to form triangles is not negligible. The clustering coefficient of the network of modules vanishes as $\langle C\rangle \sim {M}^{-1}$ in the cases of *ϕ* = 4.0 and MMNs, while it vanishes as $\langle C\rangle \sim {M}^{-\mathrm{1/2}}$ for *ϕ* = 2.5. Hierarchically organized networks are clustered with coefficient independent of the size^44^. So, the analysis of Fig. 3 shows the lack of hierarchy in the modular networks of our investigation.

The average shortest path is defined as the average minimal graph distance among every pair of vertices^42^. For the presented modular networks, this increases logarithmically with the size as shown in Fig. 3(b) and Table 1. So, the investigated networks have infinite dimension besides the lack of hierarchy.

**Table 1:** Logarithmic regressions for the average shortest distance in modular networks.

	Vertex networks	Module networks
MMN	〈*l*〉 = 2.35 + 3.19ln(*M*)	〈*l*〉 = −0.22 + 0.71ln(*M*)
*ϕ* = 4.0	〈*l*〉 = 3.67 + 2.88ln(*M*)	〈*l*〉 = 0.37 + 0.61ln(*M*)
*ϕ* = 2.5	〈*l*〉 = 7.91 + 1.35ln(*M*)	〈*l*〉 = 1.73 + 0.272ln(*M*)

We analyzed both the original vertex network and the one where modules are considered as vertices connected only by the intermodular edges. Correlation coefficient of the regressions is *r*^2^ > 0.999 for MMN and *ϕ* = 4.0, while *r*^2^ = 0.99 for *ϕ* = 2.5.

### Epidemic process on modular networks

We ran time dependent simulations of SIS dynamics, in which an infected (active) vertex *i* spontaneously heals (inactivates) with rate *μ*_*i*_ and infects each of its susceptible nearest neighbors with rate *λ*_*i*_. We used the statistically exact and optimized Gillespie algorithm detailed elsewhere^45^ with different initial conditions: decay from fully infected initial states and spreading simulations started from a single infected vertex^46^; see Methods’ section. Finite size effects were investigated using networks of size *N* ≈ *M*〈*S*_*g*_〉 with *M* = 10^3^, 10^4^ and 3×10^4^ modules, remembering that 〈*S*_*g*_〉 = 10^3^ was adopted in all cases.

In the SIS dynamics, the transition point is governed by the long-term self activation of hubs and their mutual reactivation through connected paths^47–49^, such that it presents a null threshold in case of PL networks $P(k)\sim {k}^{-\gamma }$ in the infinite size limit with ${k}^{[{\rm{upp}}]}\to \infty$. In order to deal with a finite threshold in the thermodynamic limit, we considered two types of disorders called hereafter *topological* or *intrinsic* disorder. The vertex degrees are distributed according to a truncated PL with ${k}^{[{\rm{low}}]}=3$, ${k}^{[{\rm{upp}}]}=58$ and *γ* = 2.7 in the case of topological disorder. Infection and healing rates *λ*_*i*_ = *λ* and *μ*_*i*_ = 1 (fixing the time scale) are uniform for all edges and vertices, respectively, and the disorder is due to vertex degree variability. We considered *P*(*k*) = *δ*_*k*,4_ for the intrinsic disorder case, such that each module forms a random regular network (RRN)^50^, in which every vertex has the same degree but the connections are random. Since topological disorder is negligible in this RRNs, the intrinsic disorder is introduced in the healing rates *μ*_*i*_ of each vertex *i* that take binary values 1 − *ε* or 1 + *ε* with equal chance, while the infection rate is still uniform with *λ*_*i*_ = *λ*. Note that, when investigated on homogeneous degree networks such as RRNs, the SIS is equivalent to the contact process^51^ used in previous studies of GPs on networks^22,27,52,53^.

#### Density decay analysis

**Figure 4:**
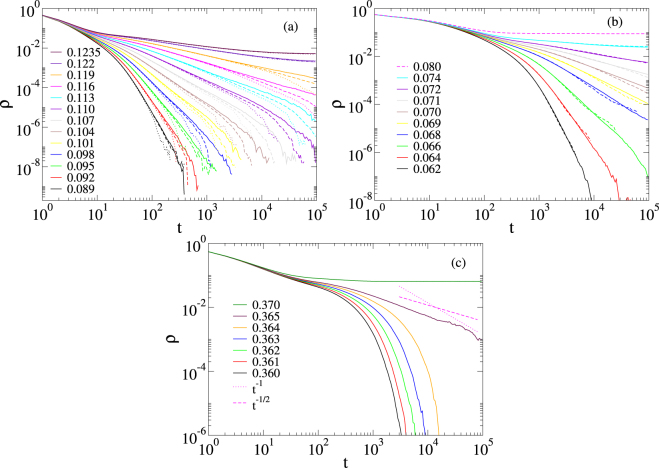
Density decay on a single MMN. (**a**) Decay analysis for SIS with topological disorder introduced by a degree distribution $P(k)\sim {k}^{-2.7}$ and ${k}^{[\text{upp}]}=58$. The numbers of modules are *M* = 10^3^ (dotted lines), *M* = 10^4^ (dashed lines), and *M* = 3 × 10^4^ (solid lines). Legends indicate the values of *λ*. (**b**) Decay analysis for SIS with intrinsic disorder (*ε* = 0.9) on MMNs of sizes *M* = 10^3^ (dashed lines) and *M* = 10^4^ (solid lines) where the modules are themselves RRNs. (**c**) SIS decay without intrinsic disorder (*ε* = 0) on a single MMN of *M* = 10^3^ modules, each one consisting of a RRN.

We show the density decays for a given realization of a MMN for three models of disorder in Fig. 4. Similar results were found for the other analyzed network realizations (up to 20). For the topological disorder, a finite size analysis increasing the number of modules is presented in Fig. 4(a). The curves reveal non-universal PLs in the 0.089 ≤ *λ* < 0.12 extended region, which do not change within statistical error margins as the number of modules increases from *M* = 10^3^ to 3×10^4^. Thus, contrary to the case of SIS on non-modular PL networks^30^, we see a GP behavior. It is important to mention that the critical regimes hold for intermediate times since the networks are still finite. Furthermore, the analysis provides numerical evidences that the transition point is also size independent. The case of strong intrinsic disorder given by *ε* = 0.9, shown in Fig. 4(b), also presents extended region of critical behavior with non universal PLs preserved as the sizes are increased. It is worth noting that the SIS dynamics on MMNs without intrinsic nor topological disorder (*μ*_*i*_ = 1), shown in Fig. 4(c), does not show GPs and the critical behavior is given by $\rho \sim {t}^{-\mathrm{1/2}}$, instead of a regular mean-field decay^20^ $\rho \sim {t}^{-1}$. This was also found in generalized small-world networks for which the GP shrank to a very narrow region^52^. We also investigated weaker intrinsic disorder using *ε* = 0.5 and observed GPs in several networks realizations, but in others they were weak or absent. However, when we performed disorder realization averaging, GPs became evident for both values of *ε* (see Supplementary Information for figures).

**Figure 5:**
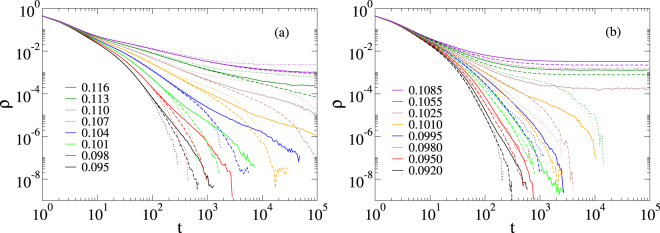
Density decay for SIS with topological disorder on a single PMN. The module size distributions with exponents (**a**) *ϕ* = 4.0 and (**b**) *ϕ* = 2.5 are shown. The finite size analysis is done using *M* = 10^3^ (dotted lines), 10^4^ (dashed lines), and 3×10^4^ (solid lines). Other network parameters are given in text and the values of *λ* indicated in the legends.

We investigated the effects of module size variability considering PMNs with topological disorder only. These exhibit the same truncated PL for degree distribution and average sizes of modules as those of MMNs to permit a comparison. In Fig. 5(a), we show extended regions of *λ* with PL tails in the density time decays for *ϕ* = 4.0, which corresponds to a heterogeneous, but finite variance distribution. These results look qualitatively similar to those of the MMN case. Finite size effects are stronger, but a GP occurs in the interval 0.095 < *λ* < 0.115, which is narrower than in the monodisperse case. Noticeably, GPs are not observed for the scale-free case with *ϕ* = 2.5 shown in Fig. 5(b), in which modules of essentially every size appear. A finite variance of *Q*(*S*_*g*_) reduces the RR effects in comparison with MMNs, since some large modules have many intermodular connections ${k}^{[{\rm{out}}]}\gg \langle {k}^{[{\rm{out}}]}\rangle$ reducing their independence. For an infinite variance the situation becomes drastic. A single module can contain a considerable large fraction of the whole network and alone rules the critical dynamics of the system becoming equivalent to the non-modular case^30,54,55^. Once we have established under which conditions of module size variability the GPs are robust, in the rest of the paper we consider only the case of MMNs, stressing that the central conclusions are the same as in the case of a finite variance in the module size distribution.

#### Spreading analysis

**Figure 6:**
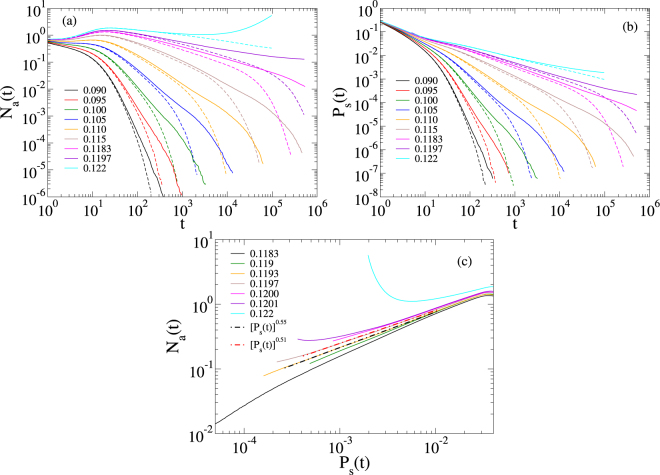
Spreading analysis for SIS on a single MMN. Only topological disorder is introduced as a truncated PL $P(k)\sim {k}^{-2.7}$ with ${k}^{[\text{upp}]}=58$. Finite size analyses of the (**a**) number of active nodes and (**b**) survival probability are done with *M* = 10^3^ (dashed lines) and 10^4^ (solid lines) modules. (**c**) Determination of the transition point in a double logarithmic plot of *N*_a_(*t*) vs *P*_s_(*t*) for *M* = 10^4^ modules. Infection rates are indicated in the legends.

Figure 6(a) shows the number of active vertices as a function of time in spreading simulations on a MMN. For regular dynamical criticality, this quantity is expected to evolve as *N*_a_(*t*) ∝ *t*^*η*^. One can see non-universal PL tails in a range similar to the one found in the density decays, including a similar exponential cutoff for long times due to the finite size of the networks. The survival probability curves, *P*_s_(*t*), exhibit a very similar behavior [Fig. 6(b)] with the same exponents as those of the *ρ*(*t*) decays at a given *λ* within the critical region, expressing that the rapidity reversal symmetry^20,56^ is unbroken by the quenched disorder. This symmetry implies, for example, that the asymptotic probability (*t* → ∞) to find one infected vertex at a randomly chosen location is weakly dependent on the initial condition or, more precisely, *ρ*(*t*) ∝ *P*_s_(*t*).

Due to the extended interval with PLs and the corrections, it is hard to estimate the transition point location and the time decay functional form accurately. Simple PL fitting results in *P*_*s*_(*t*) ∝ *t*^−*δ*^ with *δ* = 0.42(1) at ${\lambda }_{{\rm{c}}}\simeq 0.12$. Assuming a scaling in the form ${P}_{s}(t)\sim \,\mathrm{ln}\,{(t/{t}_{0})}^{-\tilde{\delta }}$, as in case of the absorbing state phase transition with strong disorder in lower dimensions^57^, we could obtain $\tilde{\delta }\approx 5$. Neither of these is in agreement with the regular mean-field behavior obtained for absorbing state phase transitions with quenched disorder in high dimensions^58^. We applied an alternative method^52^, which assumes that the leading correction to the scaling comes from the same scale *t*_0_ in the critical behaviors of *P*_s_(*t*) and *N*_a_(*t*). Plotting ln[*N*_a_(*t*)] against ln[*P*_s_(*t*)], transition point curves must fit on a straight line. As Fig. 6(c) shows, this allows an estimate for the transition point *λ*_c_ = 0.1195(2), in which the slope is 0.53(2).

**Figure 7:**
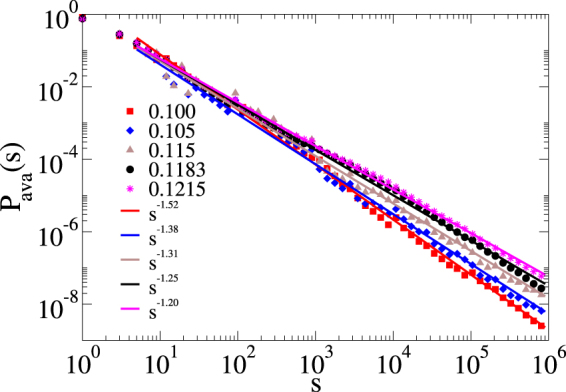
Avalanche size distributions of the SIS spreading on a single MMN. Only topological disorder is considered and the number of modules is *M* = 10^3^. Different values of *λ* are indicated by the legends. Simple PL tail fits are also shown.

We also determined the avalanche size distributions *P*_ava_(*s*) in spreading simulations. The size of an avalanche is defined as the total number of sites *s* activated during a spreading experiment. The results for *M* = 10^3^ can be seen in Fig. 7. Power-law behavior occurs for the 10 < *s* < 10^6^ region with a variation of the exponent as a function of *λ*. A PL fitting for the 0.1 ≤ *λ *≤ 0.1215 region results in *P*_ava_(*s*) ∝ *s*^−*τ*^ with 1.20 ≤ *τ *≤ 1.52, which encloses mean-field exponent of the directed percolation class (*τ* = 3/2)^59^. Curiously, this mean-value is consistent with reports for activity avalanches observed in the brain^6,12^. However, it is important to remember that other mechanisms can explain the exponent^5^ *τ* = 3/2. On the other hand, since the spreading and decay exponents depend on *λ*, the same should happen for *τ*, as the consequence of the scaling relation *τ* = (1 + *η* + 2*δ*)/(1 + *η* + *δ*) for absorbing state phase transitions^59^. Indeed, the 3/2 exponent also appears in the avalanche mean-field exponents of many models and several universality classes (DP, Dynamical Percolation, Random Field Ising Model, etc.)^56^. However, in our case, the universality class seems to be different, since the 3/2 exponent is found within the GP, while at the critical point *λ*_*c*_ ≈ 0.12 the measured *τ* is smaller.

## Discussion

We have analyzed the activity spreading of the SIS model in loosely connected, non-hierarchical modular networks and observed extended regions of non-universal scaling behavior for intrinsic [Figs 4(a), 5(a) and 6] and topological [Fig. 4(b)] disorders, using density decay and spreading analysis. The interval of the control parameter with dynamical critical region, which was robust under finite size analysis, showed size similar to previous studies on hierarchical networks^26–28^. It depends on the disorder type and shrinks only in case of a module size distribution of diverging variance [Fig. 5(b)]. The time window where we observed scaling undergoes an exponential cutoff. The reason is that we are dealing with infinite dimensional networks, for which increasing the number of vertices by one order of magnitude increases the diameter only by a few unities as shown in Fig. 3(b). However, the range of the scaling regime is improved with the size, as can clearly be read off from Fig. 4(a). This is clarified further via the local slope analysis in the Supplementary Information. However, the time window size of power laws increases modestly for non MMNs. Note that logarithmic corrections are common in GPs and this can really be observed by our local slope analysis shown in the Supplementary Information. Finally, the presented scaling regimes correspond to single network realizations, while the power-law regime is increased if averages are performed over many independent networks as exemplified in the Supplementary Information.

At a first glance, the results presented up to this point are in odds with the conjecture that infinite dimensional networks cannot sustain real GPs^22^. Strictly speaking, one may argue that the finite module sizes in the monodisperse case imply that RR lifespans can be huge, but bounded and thus the observed PLs correspond to very strong Griffths effects, differing from real GPs in the sense that they disappear in the thermodynamic limit. However, we also found size-independent GPs in the polydisperse case with *ϕ* = 4.0, where this size restriction does not apply. To understand this, we express the typical *S*_*c*_ for which at least one module of size *S* > *S*_*c*_ is present as

1 $$M{\int }_{{S}_{c}}^{{\rm{\infty }}}Q(S)dS\sim 1\Rightarrow {S}_{c}\sim {M}^{1/(\varphi -1)},$$

implying that the sizes of the largest modules diverge as their number is increased. The same result can be deduced more rigorously using extreme-value theory^60^.

**Figure 8:**
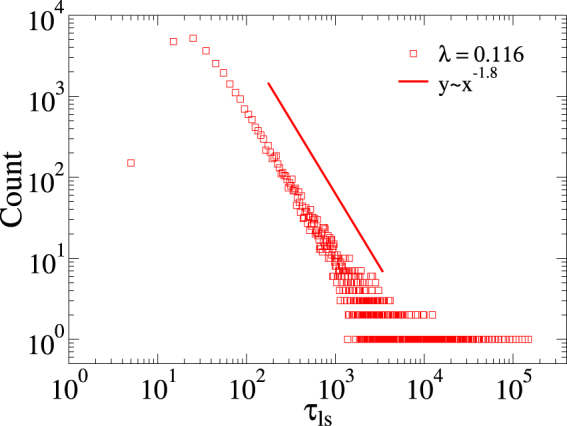
Distribution of activity lifespan within individual modules. Distribution of the activity lifespan *τ*_ls_ for SIS dynamics computed in individual modules of a single MMN with *M* = 3×10^4^ and *λ* = 0.116 inside the GP. The distribution is computed by binning time windows of size Δ*τ*_ls_ = 10.

The underlying mechanism behind GPs is the emergence of RRs related to the nature of the activity spreading of the SIS model and to the loose intermodular connectivity of the network. Let us discuss this in the case of topological disorder, but similar reasoning can be applied for the intrinsic one too. Activity of the SIS dynamics is concentrated in localized (sub-extensive) domains, which can be hubs^29,31,61^ or densely connected groups of vertices in the innermost core of the network^39,62^. However, bridges among modules in the investigated model are randomly built, implying that the probability of being connected through highly active regions of different modules is very small. This is associated with the variability of module properties, caused by the randomness. This produces broad activity lifespan distributions in individual modules inside the GP, *λ*_0_ < *λ* < *λ*_*c*_, as shown in Fig. 8 for a MMN. In this case, the distribution follows $P({\tau }_{{\rm{ls}}})\sim {\tau }_{{\rm{ls}}}^{-a}$, with an exponent *a* ≈ 1.8. To obtain this law we computed the density of infected vertices as function of time in each module. The results could be fitted with an exponential decay $\rho \sim \exp (\,-\,t/{\tau }_{{\rm{ls}}})$ in the range *t* > 20 and we could extract the characteristic time *τ*_ls_ of each module individually. Active sites in a large fraction of these modules are therefore short lived and, from the point of view of the spreading process, behave as if they were removed. The remaining network that sustains the long-term activity can be approximated by isolated or weakly coupled patches, providing an effective zero dimensional substrate for the activity spreading. However, it must be stressed that there exist intermodular interactions, that change the decay profiles in comparison with the isolated module case (see Supplementary Information for plots).

To summarize, our analysis reveals the existence of stable GPs on small-world, thus infinite dimensional substrates, conditioned to be sparsely connected in a modular structure, as an alternative for the origin of criticality on modular systems. The hierarchical modular networks, where GPs were previously observed^26,27^ are loosely connected. Hierarchy plays an important role, by increasing the distances between the modules, thus enhancing GPs, but it is not a necessary condition. The brain criticality hypothesis via GPs raised by Moretti and Muñoz^26^ is strengthened by our results since building connectome networks^10,34,63^ is far from of being trivial and the hypothesis of hierarchy in the modular organization of the brain is not fully accepted. Especially the very restrictive condition of finite dimension is fragile due to the presence of long-range connections. The model we used, conceived to be simple, allowed us to address this specific issue which would not easily be accessed in a real brain network. We expect that our results, which were not conceived for a specific system, will be important for the investigation of criticality in other modular systems beyond brain networks. In the future, one should investigate real networks with the aforementioned properties and build models with more realistic features, such as correlation patterns^64^, exerting significant influence on the results.

## Methods

### Generation of synthetic modular networks

The network is generated as follows:

(i) The number of stubs of each vertex is drawn according to the degree distribution *P*(*k*).
(ii) Two stubs are randomly chosen. If they belong to the same group, a new edge is formed. If not, an edge is formed only if the maximal number of intermodular connections in both groups is not exceeded.
(iii) Multiple or self-connections are forbidden.
(iv) The process is iterated until all stubs are connected or it becomes impossible to form new edges without multiple or self-connections.
(v) The unconnected stubs are removed. We study only the giant component which, in the present studies, contains almost all vertices of the network.

The number of removed stubs is a tiny fraction (less than 0.02% of the stubs) and does not play any relevant role on the network properties shown in Fig. 3 or Table 1.

### Network metrics

The modularity coefficient is defined by^42^

2 $${Q}_{{\rm{m}}{\rm{o}}{\rm{d}}}=\frac{1}{N\langle k\rangle }\sum _{ij}({A}_{ij}-\frac{{k}_{i}{k}_{j}}{N\langle k\rangle })\delta ({g}_{i},{g}_{j}),$$

where *A*_*ij*_ is the adjacency matrix, defined as *A*_*ij*_ = 1, if vertices *i* and *j* are connected and *A*_*ij*_ = 0 otherwise; *δ*(*i*, *j*) is the Kronecker delta function and *g*_*i*_ corresponds to the community that vertex *i* belongs to. For ${k}^{[\mathrm{out}]}\ll M$, we find *Q*_mod_ ≈ 1, confirming the expected modular structure of the investigated synthetic networks.

The Watts-Strogatz clustering coefficient of a vertex *i* is defined as^42^

3 $${C}_{i}=\frac{{e}_{i}}{{k}_{i}({k}_{i}-1)/2},$$

Where *e*_*i*_ is the number of edges interconnecting the *k*_*i*_ nearest neighbors of node *i*. The average clustering coefficient is a simple average over all vertices of the network.

### Computer implementation of the SIS model

Statistically exact simulations of the SIS dynamics in a network with infection and healing rates *λ* and *μ* can efficiently be simulated using pseudo-process method described elsewhere^45^. A list with all infected vertices, their number *N*_inf_ and the number of edges *N*_e_ emanating from them are recorded and constantly updated. In each time step, we proceed as follows.

(i) With probability
  4 $$p=\frac{\mu {N}_{{\rm{\inf }}}}{\mu {N}_{{\rm{\inf }}}+\lambda {N}_{{\rm{e}}}}$$
  an infected vertex is selected with equal chance and healed.
(ii) With complementary probability 1−*p*, an infected vertex is selected with probability proportional to its degree. A neighbor of the selected vertex is chosen with equal chance and, if susceptible, it is infected. Otherwise no change of state happens and the simulation runs to the next time step.
(iii) The time is incremented by

5 $$\tau =-\,\frac{\mathrm{ln}(u)}{\mu {N}_{{\rm{\inf }}}+\lambda {N}_{{\rm{e}}}}$$

where *u* is a pseudo random number, uniformly distributed in the interval (0, 1).

For each network realization averages were computed over 100 to 500 independent dynamic runs in the decay simulations, started with all vertices infected. For spreading analyses, which begins with a single infected vertex, the process is started 10 to 100 times at each vertex of the network.

### Data Availability

The datasets generated during and/or analysed during the current study are available from the corresponding author on reasonable request.

## Electronic supplementary material

Supplementary Information
