# Supplementary material for: Griffiths phases in infinite-dimensional, non-hierarchical modular networks
Source: Sci Rep. 2018 Jun 14;8:9144. doi: 10.1038/s41598-018-27506-x (PMC6002411; doi:10.1038/s41598-018-27506-x)
Supplement: Supplementary file 1 — Supplementary Information [file 41598_2018_27506_MOESM1_ESM.pdf]

Supplementary Information for "Griffiths phases in infinite-dimensional, non-hierarchical modular networks"

Wesley Cota,[1] Géza Ódor,[2] and Silvio C. Ferreira[1,3]

[1] *Departamento de Física, Universidade Federal de Viçosa, 36570-000, Viçosa, Minas Gerais, Brazil*
[2] *MTA-MFA-EK, Center for Energy Research of the Hungarian Academy of Sciences, H-1121 Budapest, P.O. Box 49, Hungary*
[3] *National Institute of Science and Technology for Complex Systems, Brazil*

We present complementary figures for decay analyses.

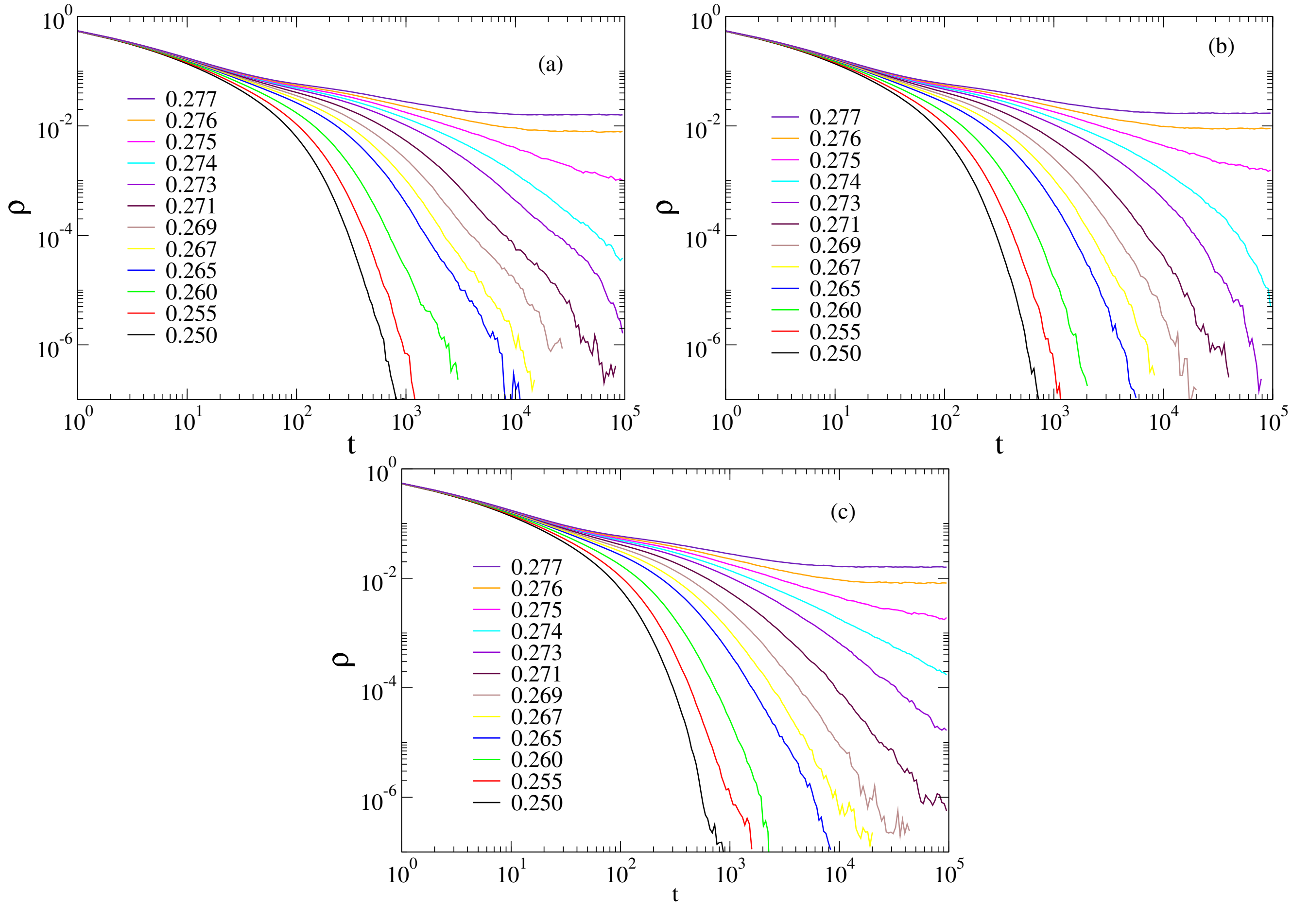

FIG. SI-1. SIS decay with intrinsic disorder of the strength $\varepsilon = 0.50$ on a single MMN with $M = 10^3$ modules and with uniform degree distribution $P(k) = \delta_{k,4}$ (modules are random regular networks). The infection rates are shown in the legends. We show two different disorder realizations, fixed for all dynamical samples, which does (a) or does not (b) exhibit GP. (c) When a new disorder realization is generated for each dynamical sample, the average leads to GP on extended scaling law regions in the interval $0.265 \lessapprox \lambda \lessapprox 0.275$. 100 to 500 dynamical runs were used to compute averages.

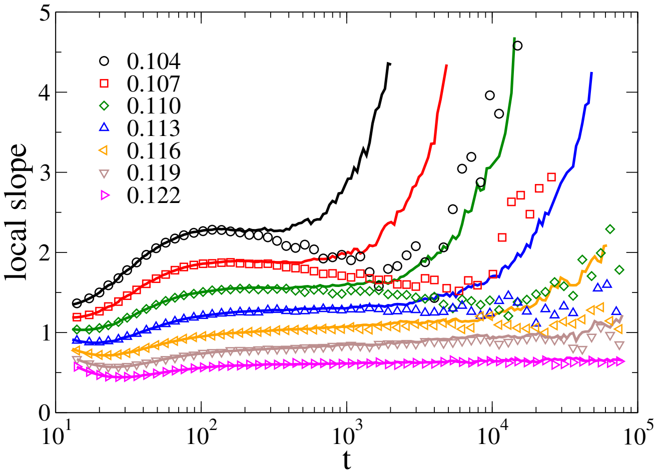

FIG. SI-2. Local exponent analysis for the density decay curves on MMN with sizes $M = 10^4$ (lines) and 3×10^4 (symbols) modules. The local exponent is defined as $\alpha_{\rm eff} = -d(\ln \rho)/d(\ln t)$. Following an interval of low variation the local exponents become very large, indicating an exponential decay. As the number of modules increases the exponential tails emerge at later times.

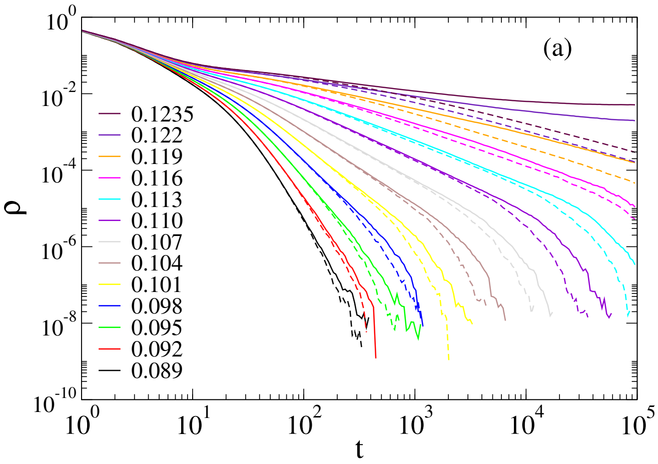

FIG. SI-3. Comparison of SIS decays for interconnected and disconnected MMNs with $M = 10^4$ modules. The disconnected case with $k_g^{[\rm out]} = 0$ are presented by dashed lines and the interconnected one with $k_g^{[\rm out]} = 5$ is represented by solid lines. The other network parameters are $S = 10^3$, $\gamma = 2.7$, $k^{[\rm low]} = 3$, and $k^{[\rm upp]} = 58$. The values of λ are indicated in the legend. The same color correspond to the same λ. 100 to 500 dynamical runs were used to compute averages. It can be clearly seen that the interconnected case have higher activity than the disconnected one due to the long-range activation mechanism of the SIS dynamics [1–3] that permits long-range dynamical interactions between vertices at different modules.

[1] M. Boguñá, C. Castellano, and R. Pastor-Satorras, Phys. Rev. Lett. **111**, 068701 (2013).
[2] S. Chatterjee and R. Durrett, Ann. Probab. **37**, 2332 (2009).
[3] S. C. Ferreira, R. S. Sander, and R. Pastor-Satorras, Phys. Rev. E **93**, 032314 (2016).
